# Supplementary material for: Machine learning-assisted decoding of temporal transcriptional dynamics via fluorescent timer
Source: Nat Commun. 2025 Jul 1;16:5720. doi: 10.1038/s41467-025-61279-y (PMC12219120; doi:10.1038/s41467-025-61279-y)
Supplement: Supplementary file 1 — Supplementary Information [file 41467_2025_61279_MOESM1_ESM.pdf]

Supplementary Information for

Machine Learning-Assisted Decoding of Temporal Transcriptional Dynamics via Fluorescent Timer

This file includes the following Supplementary Figures and Tables:

- Supplementary Figure 1
- Supplementary Figure 2
- Supplementary Figure 3
- Supplementary Figure 4
- Supplementary Figure 5
- Supplementary Figure 6
- Supplementary Table 1
- Supplementary Table 2
- Supplementary Table 3
- Supplementary Table 4

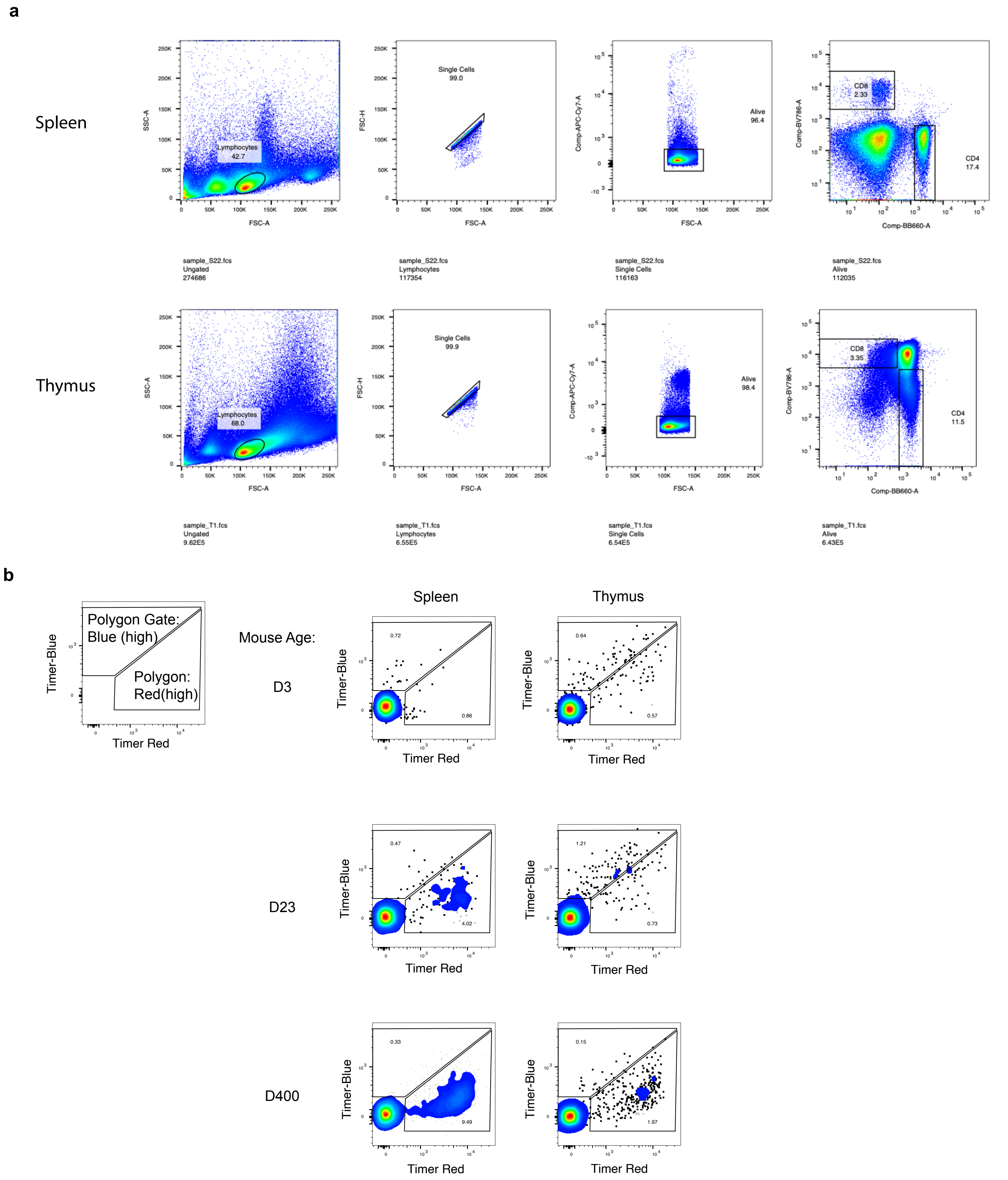

Supplementary Figure 1. Gating strategies used in this study.
(a) Successive gates for identifying splenic CD4+ T cells, thymic CD4-single positive cells.
(b) Polygon gates defining ‘Polygon-Blue (high)’ and ‘Polygon-Red (high)’ Foxp3 Timer+ cells.

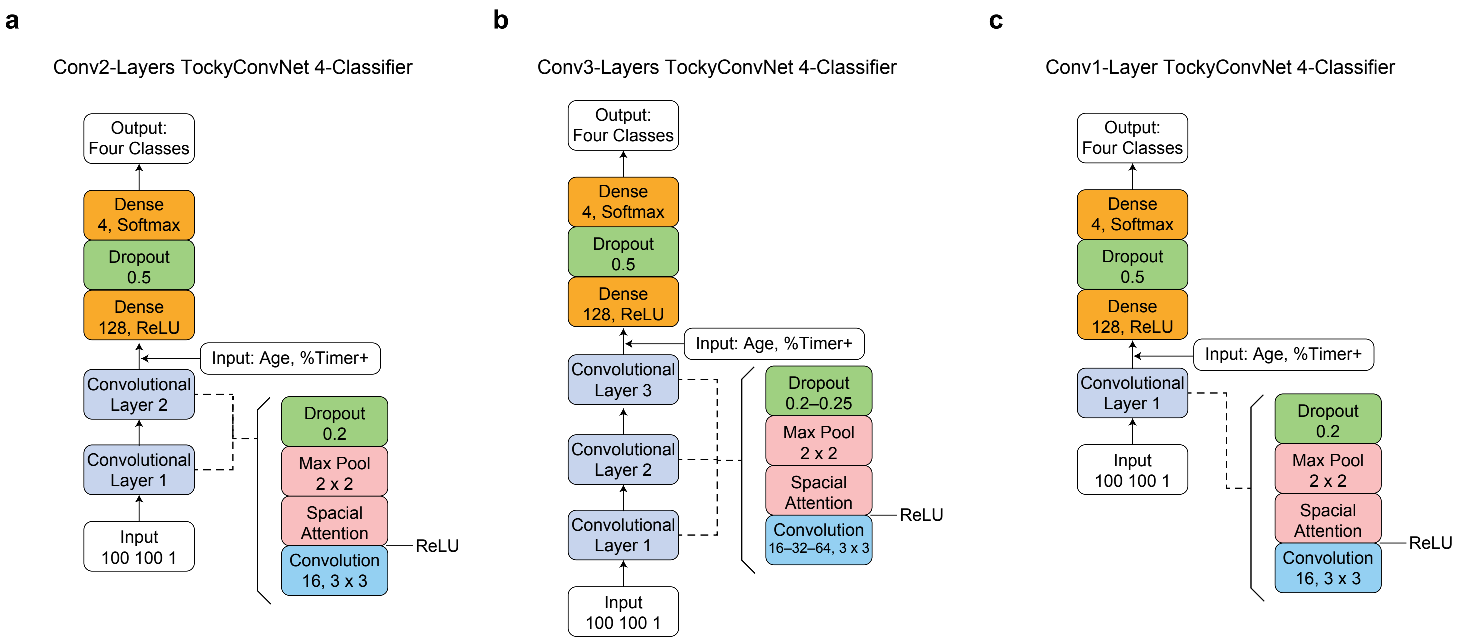

Supplementary Figure 2. Convolutional neural network architectures used for Foxp3 Timer Neonatal-to-Ageing benchmarking data.

All convolutional neural network architectures used for the benchmarking data are shown.

(a) Model with 2 convolutional layers (Conv2-layer model) for four-class classifier.

(b) Model with 3 convolutional layers (Conv3-layer model) for four-class classifier (the same model as shown in Figure S8a).

(c) Model with 1 convolutional layer (Conv1-layer model) for four-class classifier.

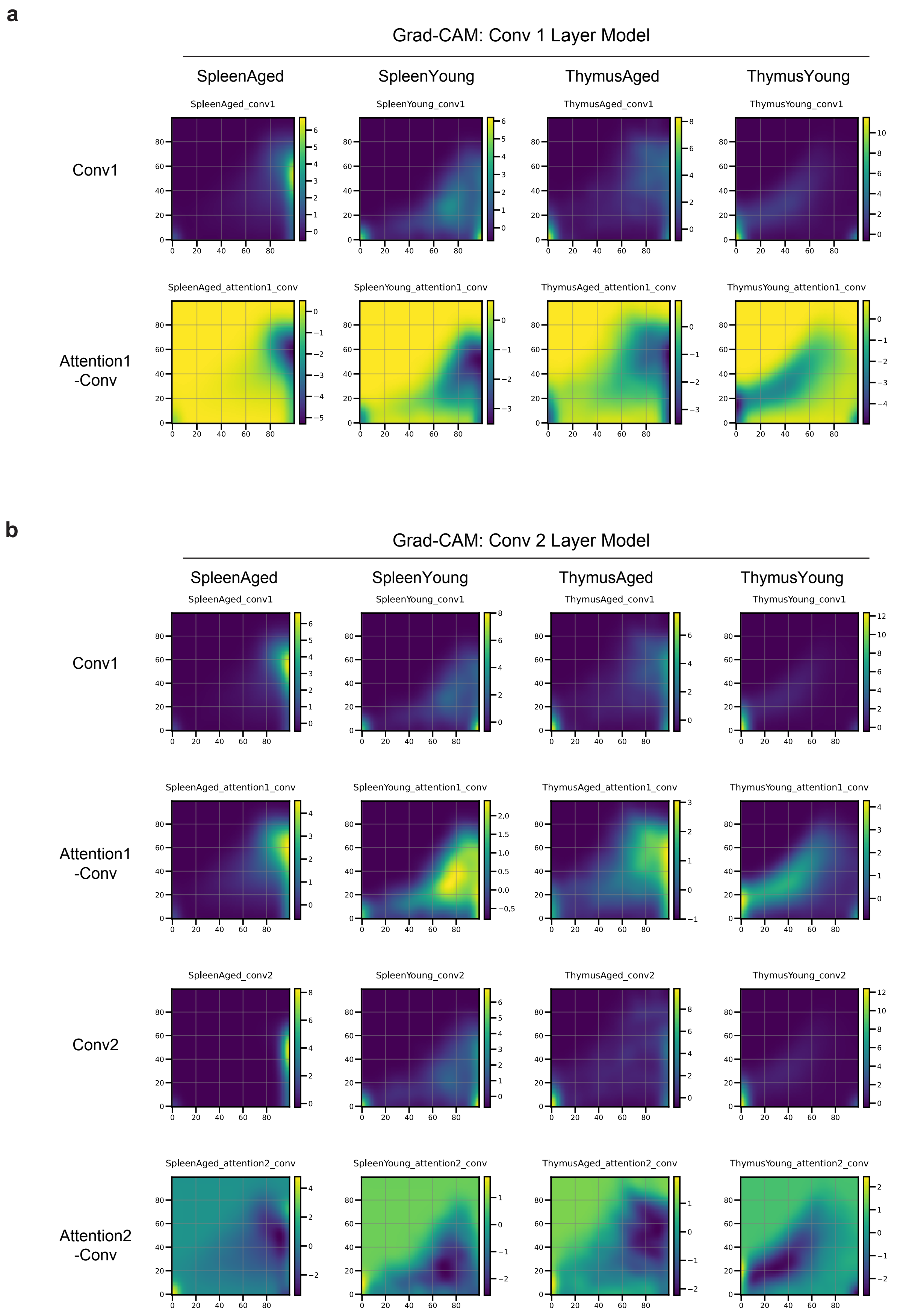

Supplementary Figure 3. Gradient-weighted Class Activation Mapping (Grad-CAM) analysis of convolutional neural network models with different convolutional layer numbers.
Grad-CAM analysis for the four classes was performed for each of the convolutional layers from (a) Conv1-layer model and (2) Conv2-layer model.
The model architectures are detailed in Supplementary Figure 2.

a

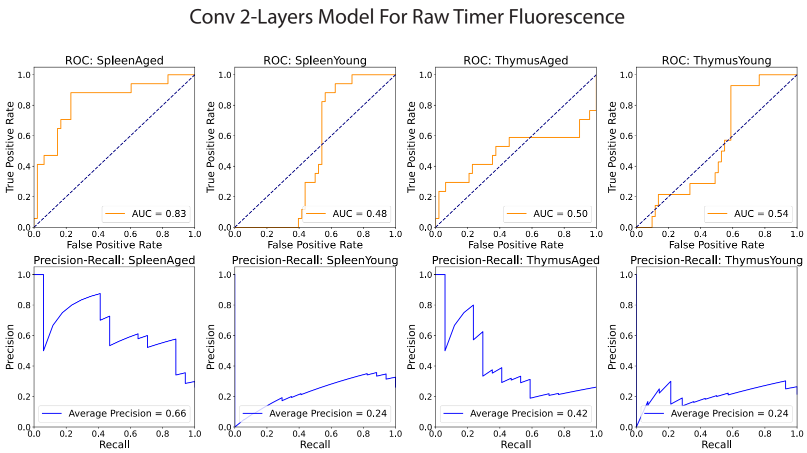

b

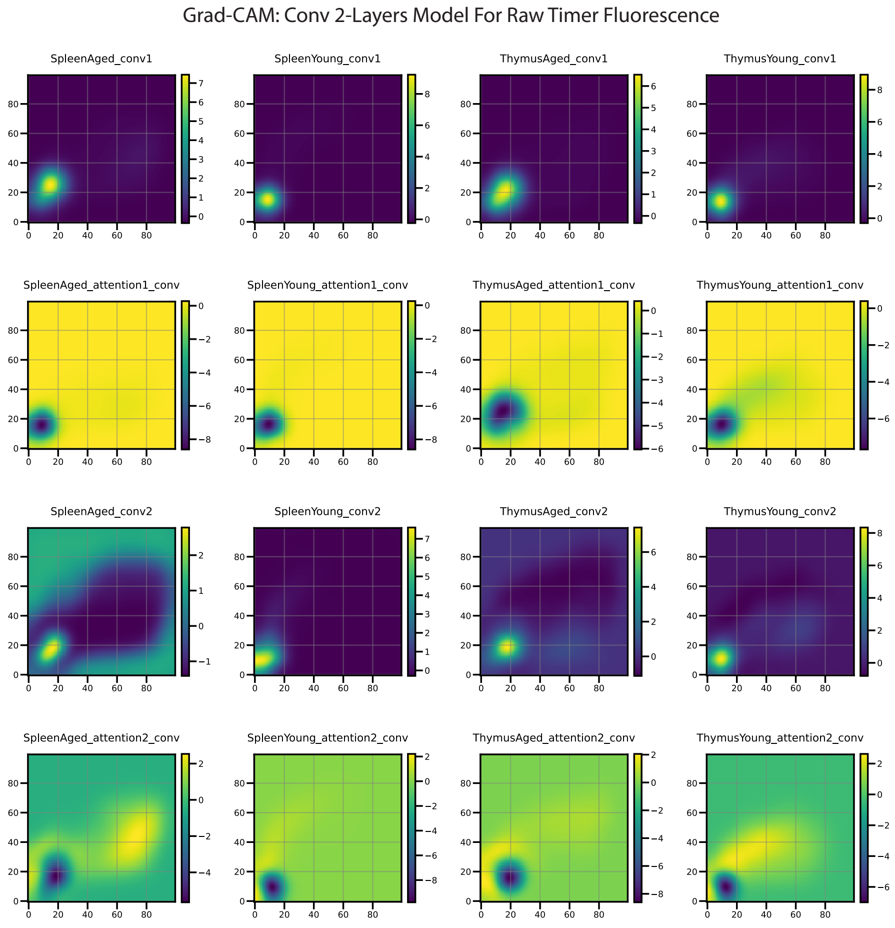

Supplementary Figure 4. Effects of Fluorescent Timer data preprocessing on model performance. The Conv2-layer, four-class classification model (Supplementary Figure 2a) was trained using raw Timer fluorescence data without preprocessing.
(a) Receiver operating characteristic (ROC) analysis.
(b) Grad-CAM analysis for four classes.

a

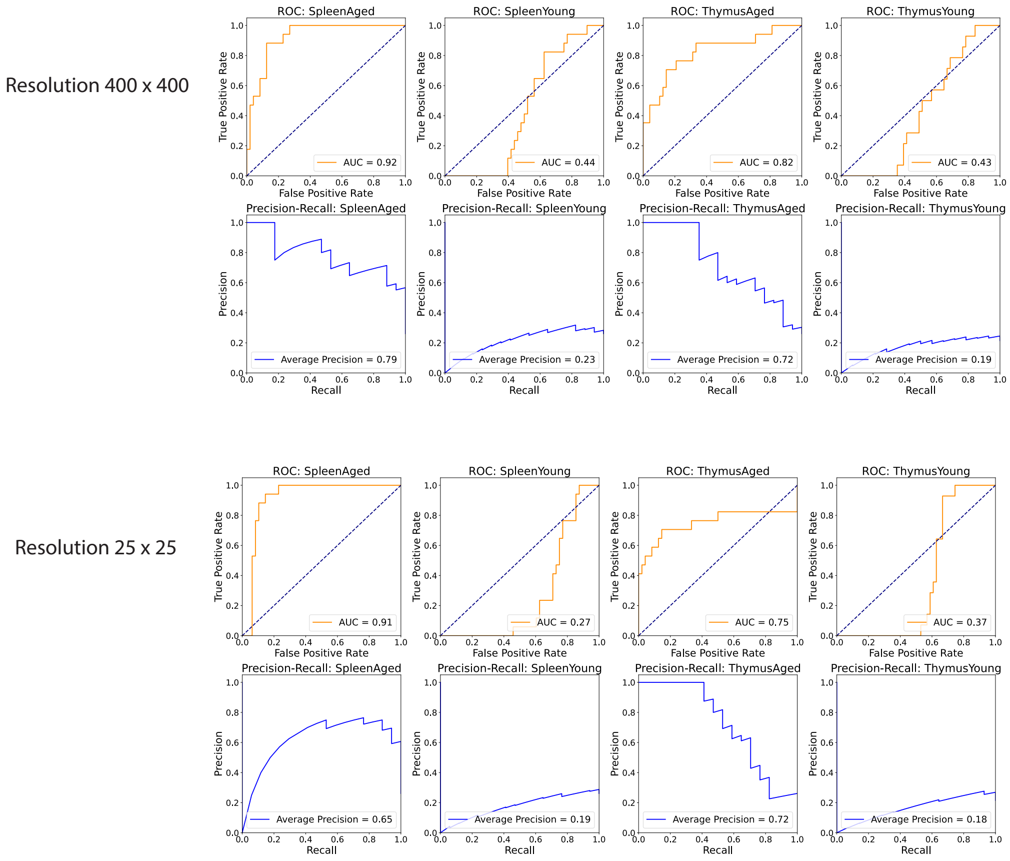

b

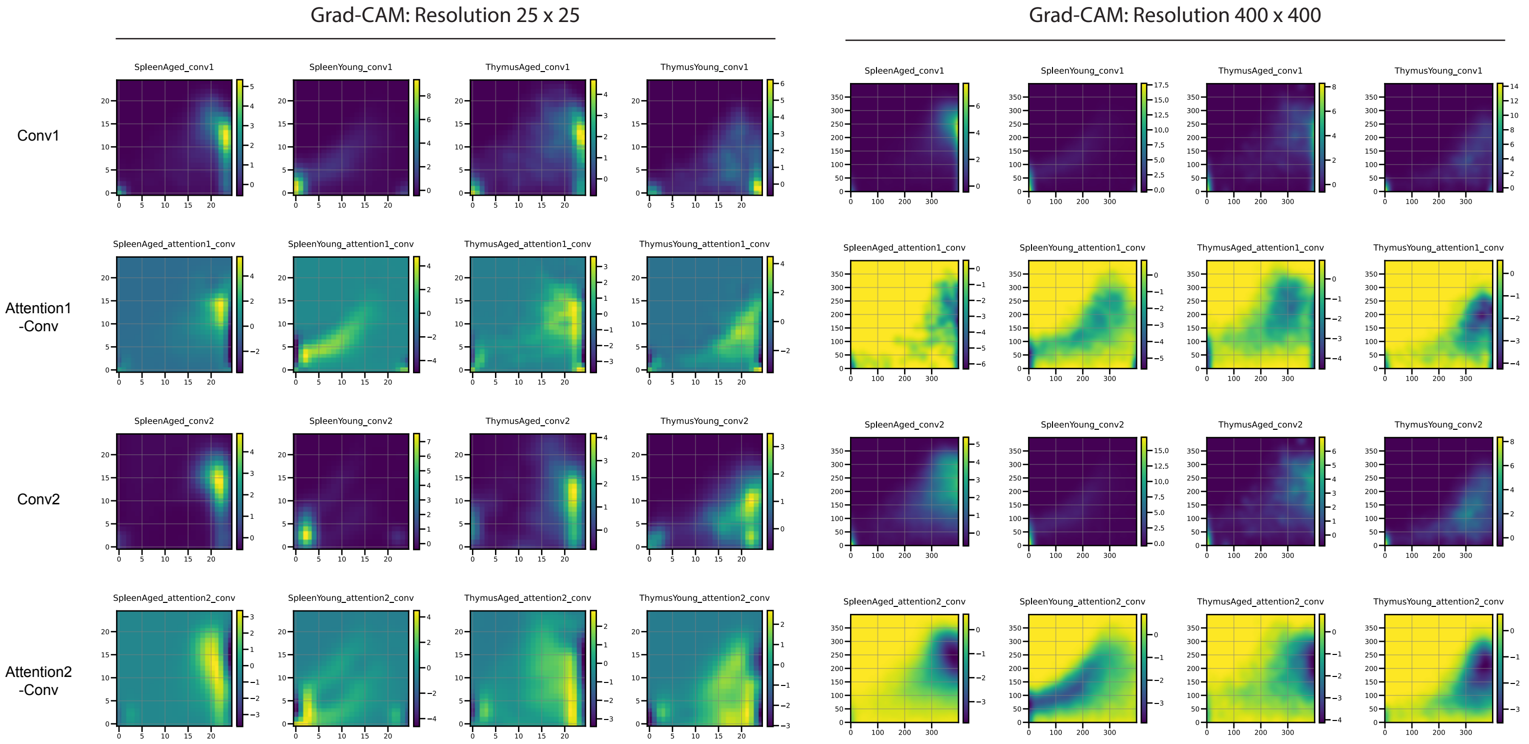

Supplementary Figure 5. Impact of data resolution on model performance.
Performance of the Conv2-layer, four-class classification model (Supplementary Figure 2a) was evaluated at resolutions from low (25×25) to high (400×400).
(a) ROC analysis.
(b) Grad-CAM analysis for four classes.

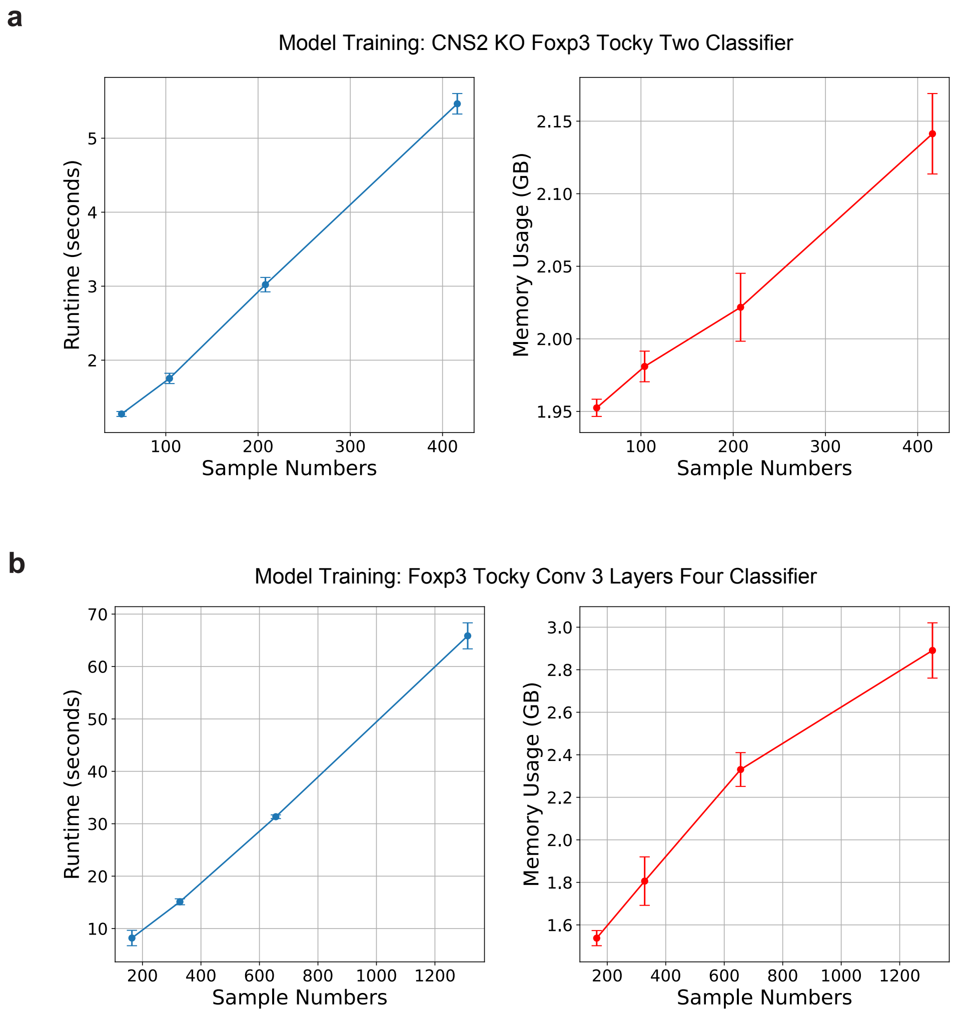

Supplementary Figure 6. Computational resource analysis using TockyConvNet models. Computational runtime (a) and memory usage (b) were assessed across increasing dataset sizes for major TockyConvNet models used in this study. Error bars represent standard deviations.

Supplementary Table 1. Datasets generated and analysed in the current study.
(a) Overview of Datasets.
(b) Sample Numbers in Foxp3-Tocky Neonatal-to-Ageing training dataset.

(a) Overview of Datasets

Dataset	Genotype	Age Range	Sample Number	Note
CNS2 KO Foxp3 Tocky Training Data	Foxp3 Timer (CNS2KO),	W7	14 WT and 20 KO	Two independent experiments were pooled
CNS2 KO Foxp3 Tocky Test Data	Foxp3 Timer (CNS2KO),	W7	22 WT and 27 KO	Two independent experiments were pooled
WT Foxp3 Tocky Neonatal-to-Ageing Training Data	Foxp3 Timer (WT)	D1 – W41	47 Spleen (21 Aged and 26 Young) and 56 Thymus (21 Aged and 35 Young)	Four independent experiments were pooled
WT Foxp3 Tocky Neonatal-to-Ageing Test Data	Foxp3 Timer (WT)	D1 – W22	31 Spleen (17 Aged and 14 Young) and 34 Thymus (17 Aged and 17 Young)	Seven independent experiments were pooled

(b) Sample Details of Foxp3 Neonatal-to-Ageing Training Dataset.

Age	Age Category	Organ	Number_of_Samples	Average_Cell_Number	SD_Cell_ Number	Note
D1	Young	Spleen	NA	NA	NA	Too few CD4+ T cells for analysis
D2	Young	Spleen	NA	NA	NA	Too few CD4+ T cells for analysis
D3	Young	Spleen	11	267818	138476	
D4	Young	Spleen	4	322000	129182	
D8	Young	Spleen	1	1190000	NA	
W3	Young	Spleen	9	2781111	1659597	
W5	Aged	Spleen	3	2880000	1354696	
W14	Aged	Spleen	11	4912727	1543795	
W41	Aged	Spleen	7	4777143	1900857	
D1	Young	Thymus	3	1019333	292966	
D2	Young	Thymus	6	1036000	295126	
D3	Young	Thymus	11	1632000	581047	
D4	Young	Thymus	4	1972000	557499	
D8	Young	Thymus	2	3022500	208597	
W3	Young	Thymus	9	8265556	6948729	
W5	Aged	Thymus	3	5786667	2203028	
W14	Aged	Thymus	11	8476364	3344749	
W41	Aged	Thymus	7	2937143	2300012	

Supplementary Table 2. Statistical Summary of Regression Analyses Comparing Thymus-Spleen Model Score Against Age and Logged Ag

Summary of regression analyses comparing thymus–spleen model scores against age and log-transformed age.

Model	R-squared	Adj. R-squared	F-statistic	Prob (F-statistic)	Log-Likelihood	AIC
**Thymus Data vs Age**	0.949	0.943	167.4	2.34E-12	-48.107	102.2
**Thymus Data vs log2 Age**	0.946	0.94	158.8	3.68E-12	-48.634	103.3
**Spleen Data vs Age**	0.741	0.706	21.45	3.99E-05	-50.595	107.2
**Spleen Data vs log2 Age**	0.92	0.909	85.89	6.10E-09	-40.053	86.11

Supplementary Table 3. Summary of ConvNet Models

Layer architectures and parameter counts of major TockyConvNet models.

Model	Layers	Convolutional layer blocks	Total Parameters	Figure Number
CNS2 KO Foxp3 Tocky Two Classifier	2 Conv, 2 MaxPool, 2 Dense, Attention Mechanisms	2	3.23E+05	4
WT Foxp3 Tocky Two Classifier	2 Conv, 2 MaxPool, 2 Dropout, 2 Dense, Attention Mechanisms	2	2.57E+06	7
WT Foxp3 Tocky Two Classifier (continious score)	2 Conv, 2 MaxPool, 2 Dropout, 2 Dense, Attention Mechanisms	2	2.57E+06	7
WT Foxp3 Tocky Conv 3 Layers Four Classifier	3 Conv, 3 MaxPool, 3 Dropout, 2 Dense, Attention Mechanisms	3	1.20E+06	8, 9

Supplementary Table 4. Comparison of the ML approaches in the current study with traditional manual gating methods.
Core methods, strengths, and weakness are summarised for traditional gating, TockyKmeansRF, and TockyConvNet.

Methodology	Core method	Strengths	Weakness
Manual Gating, Quadrant	Determining thrsholds for marker positivity	• Suitable for small sample sizes with ensured statistical power<br>• Accessible to users inexperienced in data analysis	• Limited to known populations; does not adapt after analysis<br>• Low resolution; equivalent to 2x2 bin analysis<br>• Time-consuming and may lack reproducibility without consistent batch analysis
Manual Gating, Polygon	Arbitrary Gating using 2D plots	• Feasible using small sample sizes with ensured statistical power<br>• Accessible to users inexperienced in data analysis	• Restricted to known populations; does not adapt post-analysis<br>• Prone to significant reproducibility issues, arbitrariness<br>• Incompatibility with a data-driven approach<br>• Time-consuming
TockyKmeansRF	TockyPrep, K-means, and Random Forest	• Analysis strategy evolves in a data-driven manner<br>• Simpler model compared to ConvNet	• Outputs clusters rather than providing single-cell resolution
TockyCNN and Grad-CAM	TockkyPrep, Image Conversion, ConvNet, and Grad-CAM	• Enables visually intuitive, high-resolution, data-driven analysis<br>• Provides single-cell resolution	• Risk of overfitting, necessitating careful model architecture and training strategies<br>• Requires high-quality, substantial datasets
