# Supplementary material for: Machine learning-assisted decoding of temporal transcriptional dynamics via fluorescent timer
Source: Nat Commun. 2025 Jul 1;16:5720. doi: 10.1038/s41467-025-61279-y (PMC12219120; doi:10.1038/s41467-025-61279-y)
Supplement: Supplementary file 2 — Description of Additional Supplementary Files [file 41467_2025_61279_MOESM2_ESM.pdf]

Description of Additional Supplementary Files

Supplementary Data 1. Exact p-values for Figures 3g, 4i–k, and 5d–e This excel file provides the exact p-values corresponding to statistical comparisons shown in figures 3g, 4i, 4j, 4k, and 5d–e.
